# Mutation discovery in mice by whole exome sequencing

**DOI:** 10.1186/gb-2011-12-9-r86

**Published:** 2011-09-14

**Authors:** Heather Fairfield, Griffith J Gilbert, Mary Barter, Rebecca R Corrigan, Michelle Curtain, Yueming Ding, Mark D'Ascenzo, Daniel J Gerhardt, Chao He, Wenhui Huang, Todd Richmond, Lucy Rowe, Frank J Probst, David E Bergstrom, Stephen A Murray, Carol Bult, Joel Richardson, Benjamin T Kile, Ivo Gut, Jorg Hager, Snaevar Sigurdsson, Evan Mauceli, Federica Di Palma, Kerstin Lindblad-Toh, Michael L Cunningham, Timothy C Cox, Monica J Justice, Mona S Spector, Scott W Lowe, Thomas Albert, Leah Rae Donahue, Jeffrey Jeddeloh, Jay Shendure, Laura G Reinholdt

**Affiliations:** 1 The Jackson Laboratory, 600 Main St, Bar Harbor, ME 04609, USA; 2 Baylor College of Medicine, Department of Molecular and Human Genetics, One Baylor Plaza R804, Houston, Texas 77030, USA; 3 Cold Spring Harbor Laboratory, One Bungtown Road, Cold Spring Harbor, NY 11724, USA; 4 Roche NimbleGen, Inc. Madison, WI 53719, USA; 5 National Center for Genome Analysis (CNAG), Parc Científic de Barcelona, Torre I, Baldiri Reixac, 408028 Barcelona, Spain; 6 Walter and Eliza Hall Institute, 1G Royal Parade, Parkville, Victoria 3052, Australia; 7 University of Washington, Department of Pediatrics, Division of Craniofacial Medicine and Seattle Children's Craniofacial Center, 4800 Sand Point Way NE, Seattle, WA 98105, USA; 8 Regeneron Pharmaceuticals Inc., 777 Old Saw Mill River Road, Tarrytown, NY 10591, USA; 9 Broad Institute of Massachusetts Institute of Technology and Harvard, 5 Cambridge Center, Cambridge, MA 02142, USA; 10 University of Washington, Department of Genome Sciences, Foege Building S-250, Box 355065, 3720 15th Ave NE, Seattle, WA 98195-5065, USA

## Abstract

We report the development and optimization of reagents for in-solution, hybridization-based capture of the mouse exome. By validating this approach in a multiple inbred strains and in novel mutant strains, we show that whole exome sequencing is a robust approach for discovery of putative mutations, irrespective of strain background. We found strong candidate mutations for the majority of mutant exomes sequenced, including new models of orofacial clefting, urogenital dysmorphology, kyphosis and autoimmune hepatitis.

## Background

Phenotype-driven approaches in model organisms, including spontaneous mutation discovery, standard *N*-ethyl-*N*-nitrosourea (ENU) mutagenesis screens, sensitized screens and modifier screens, are established approaches in functional genomics for the discovery of novel genes and/or novel gene functions. As over 90% of mouse genes have an ortholog in the human genome [1], the identification of causative mutations in mice with clinical phenotypes can directly lead to the discovery of human disease genes. However, mouse mutants with clinically relevant phenotypes are not maximally useful as disease models until the underlying causative mutation is identified. Until recently, the gene discovery process in mice has been straightforward, but greatly hindered by the time and expense incurred by high-resolution recombination mapping. Now, the widespread availability of massively parallel sequencing [2] has brought about a paradigm shift in forward genetics by closing the gap between phenotype and genotype.

Both selective sequencing and whole genome sequencing are robust methods for mutation discovery in the mouse genome [3-5]. Nonetheless, the sequencing and analysis of whole mammalian genomes remains computationally burdensome and expensive for many laboratories. Targeted sequencing approaches are less expensive and the data are accordingly more manageable, but this technique requires substantial genetic mapping and the design and purchase of custom capture tools (that is, arrays or probe pools) [4]. Targeted sequencing of the coding portion of the genome, the 'exome', provides an opportunity to sequence mouse mutants with minimal mapping data and alleviates the need for a custom array/probe pool for each mutant. This approach, proven to be highly effective for the discovery of coding mutations underlying single gene disorders in humans [6-12], is particularly relevant to large mutant collections, where high-throughput gene discovery methods are desirable.

Currently, there are nearly 5,000 spontaneous and induced mouse mutant alleles with clinically relevant phenotypes catalogued in the Mouse Genome Informatics database [13]. The molecular basis of the lesions underlying two-thirds of these phenotypes is currently unknown. For the remaining one-third that have been characterized, the Mouse Genome Informatics database indicates that 92% occur in coding sequence or are within 20 bp of intron/exon boundaries, regions that are purposefully covered by exome targeted re-sequencing. While this estimate is impacted by an unknown degree of ascertainment bias (since coding or splice site mutations are easier to find and hence reported and since many uncharacterized mutations remain so because they are understudied), we anticipated that exome sequencing would still be likely to capture a considerable percentage of spontaneous and induced mouse mutations. Therefore, to significantly reduce the time, effort, and cost of forward genetic screens, we developed a sequence capture probe pool representing the mouse exome. Here, we describe the utility of this tool for exome sequencing in both wild-type inbred and mutant strain backgrounds, and demonstrate success in discovering both spontaneous and induced mutations.

## Results and discussion

### Mouse exome content and capture probe design

The coding sequence selected for the mouse exome probe pool design includes 203,225 exonic regions, including microRNAs, and collectively comprises over 54.3 Mb of target sequence (C57BL/6J, NCBI37/mm9). The design was based on a unified, Mouse Genome Database-curated gene set, consisting of non-redundant gene predictions from the National Center for Biotechnology Information (NCBI), Ensembl and The Vertebrate Genome Annotation (VEGA) database [13]. The gene list is available at [14]. To manage the size of the probe pool and to avoid non-uniquely mappable regions, we excluded olfactory receptors and pseudogenes from the target sequence. In cases where an exon contained both UTR and coding sequence, the UTR sequence was included in the design. Two DNA probe pools, alpha and beta prototypes, were ultimately designed and tested. To maximize the uniformity of the sequencing libraries after capture, re-sequencing data from the alpha prototype design were empirically studied and used to inform a coverage re-balancing algorithm. That algorithm altered the probe coverage target ratio of a second design (beta prototype) in an attempt to decrease over-represented sequence coverage, and increase under-represented sequence coverage. The target (primary design) coordinates and the coordinates of the capture probes in the beta design are available at [15]. The summary statistics for each probe pool are shown in Additional file 1.

### Exome capture performance and optimization

**Figure 1:**
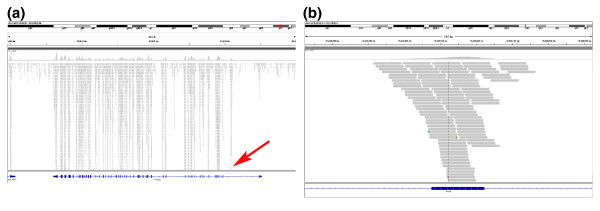
**Graphical view (Integrated Genomics Viewer) of read distribution across a gene and an exon **. **(a,b) **Gene (a) and exon (b) annotations shown are from the primary representative RefSeq annotations. The exome design encompasses a unified set of exon annotations from NCBI, Ensembl and VEGA; therefore, there are regions with high coverage, representing exons that are not shown in the primary RefSeq annotation (red arrow) but are represented in Ensembl and/or VEGA. Typical coverage across exons includes sufficient read depth to call single nucleotide variants in coding sequence and in neighboring splice acceptor and donor sites, as well as 20 to 50 bases of additional flanking intron sequence (b).

To test the alpha and beta exome probe pools and to determine whether strain background adversely influenced performance, exomes from four commonly used inbred strains (C57BL/6J, 129S1/SvImJ, BALB/cJ and C3H/HeJ) were captured and re-sequenced (Table 1). Overall, capture sensitivity was high, with just one lane of 2 × 40-bp paired-end sequencing (2 × 40 bp PE) resulting in > 96% of the targeted bases covered. The capture specificity was also high with > 75% reads mapping to targeted bases. Importantly, the sequencing data were significantly enriched, not only for coding sequence but also for flanking splice acceptor and donor sites, where deleterious mutations are frequently found (Figure 1). Genetic background only modestly impacted the sensitivity and specificity of the capture probe pools. The variation between strains was greater than within a strain (Table 1); however, the scale of the inter-strain differences observed suggests that a pool based upon exclusively the mm9 reference would be functional with any *Mus musculus *background.

**Table 1:** Direct comparison of coverage statistics from exome re-sequencing (2 × 40 bp, Illumina) of four inbred strains with two exome probe pool designs, alpha and beta

	Sample							
	
	C57BL/6J	C57BL/6J	129S1/SvImJ	129S1/SvImJ	BALB/cJ	BALB/cJ	C3H/HeJ	C3H/HeJ
Exome version	Alpha	Beta	Alpha	Beta	Alpha	Beta	Alpha	Beta
Quantitative PCR	161.81	168.53	129.43	95.75	168.92	165.08	168.38	92.00
Target exons	203,225	203,224	203,225	203,224	203,225	203,224	203,225	203,224
Target bases	54,367,346	54,367,244	54,367,346	54,367,244	54,367,346	54,367,244	54,367,346	54,367,244
Target bases covered	52,266,238	53,273,874	51,746,839	52,508,881	51,828,334	52,862,662	52,136,965	51,460,949
Percentage target bases covered	96.14	97.99	95.18	96.58	95.33	97.23	95.90	94.65
Target bases not covered	2,101,108	1,093,370	2,620,507	1,858,363	2,539,012	1,504,582	2,230,381	2,906,295
Percentage target bases not covered	3.86	2.01	4.82	3.42	4.67	2.77	4.10	5.35
Median coverage	18.45	20.74	17.93	16.37	18.05	20.75	18.76	7.86
Total reads	60,582,097	60,207,746	64,258,556	44,434,168	64,495,816	63,740,186	64,959,026	25,760,946
NC80	0.28	0.37	0.25	0.33	0.25	0.31	0.29	0.32
1/NC80	3.53	2.71	4.03	3.02	3.96	3.27	3.50	3.13

1/NC80 is the fold 80 penalty, which represents the fold of over-sequencing necessary to move 80% of the below median bases to median.

The beta design was made using a proprietary rebalancing algorithm from Roche NimbleGen (Madison, WI, USA) that removes probes from targets with high coverage and adds probes to low coverage targets in order to maximize coverage across targets. In addition to testing the beta design by exome capture and 2 × 40 bp PE Illumina sequencing of four different inbred strains, the beta design was also tested with four independent captures of C57BL/6J female DNA and sequenced on the Illumina GAII platform, 2 × 76 bp PE. The most dramatic improvement was observed in the fraction of targeted bases covered at 20× or more where the increase in uniformity resulted in 12% improvement (Additional file 2).

### Sequencing of mutant exomes

To determine the efficacy of the probe pools for mutant exome re-sequencing and mutation discovery, 15 novel mouse mutant exomes and 3 controls were captured and sequenced at multiple sites using different Illumina platforms (Illumina GAIIx, Illumina HiSeq, and both 2 × 76-bp and 2 × 100-bp PE libraries). The mutants were selected based on several parameters, including research area, mode of inheritance (dominant and recessive), strain background, and mutation type (induced and spontaneous). Where appropriate, homozygous samples were captured and sequenced (Additional file 3). In all cases, the beta exome pools provided improved capture uniformity. In the majority of cases, > 97% of targeted bases were covered by at least one read (1×). Approximately 45 million 100-bp PE reads were sufficient, on average, to provide at least 5 reads coverage of 95% of target bases (Table 2; Additional file 4), which is sufficient for detection of recessive mutations in homozygous samples. To confidently call heterozygous alleles, at least 15× coverage is preferable [4], and these data show that more than 58 million, 100-bp PE reads are likely required to obtain a minimum of 15 reads across 95% of target bases. Therefore, we anticipate that sample indexing schemes may soon enable as many as four exomes to be multiplexed per lane of an Illumina HiSeq run using the most current reagents. The raw sequencing data for mutant and inbred strains are available from the NCBI Sequence Read Archive (accession number [SRP007328]).

**Table 2:** Representative coverage statistics from exome re-sequencing (2 × 100 bp) of six mutant strains

	Sample					
	
	5330 (*hbck*)	6246 (*sunk*)	8568 (*lear*)	12856 (*shep*)	13782 (*aphl*)	13716 (*vgim*)
Targeted exons	203,224	203,224	203,224	203,224	203,224	203,224
Final target bases	54,367,244	54,367,244	54,367,244	54,367,244	54,367,244	54,367,244
Target bases covered	52,934,978	52,493,811	52,832,014	52,647,881	52,664,921	53,004,900
Percentage target bases covered	97.37	96.55	97.18	96.84	96.87	97.49
Target bases not covered	1,432,266	1,873,433	1,535,230	1,719,363	1,702,323	1,362,344
Percentage target bases not covered	2.63	3.45	2.82	3.16	3.13	2.51
Total reads^a^	39,675,108	39,641,830	31,817,686	42,405,386	59,956,764	67,359,382
Number of reads in target regions	23,319,015	23,335,916	19,211,748	25,227,205	36,227,876	39,948,582
Percentage reads in target regions	58.77	58.87	60.38	59.49	60.42	59.31
Average coverage	32.72	32.59	26.75	35.32	50.78	56.31
Median coverage	30.33	30.02	23.23	33.02	46.61	50.02
Coverage at 20×	76.4	73.6	61.9	77.5	85.8	88
Coverage at 10×	92.1	89.3	87.1	90.7	92.9	94.5
Coverage at 5×	95.7	93.8	94.3	94.4	95.1	96.2
Coverage at 1×	97.4	96.6	97.2	96.8	96.9	97.5
NC80	0.51	0.47	0.46	0.49	0.47	0.46
1/NC80	1.94	2.13	2.18	2.06	2.13	2.17

1/NC80 is the fold 80 penalty, which represents the fold of over sequencing necessary to move 80% of the below median bases to median. Coverage statistics for all samples sequenced can be found in Additional file 3. ^a^2 × 100 bp, Illumina HiSeq.

### Mapping and variant calling

Mapping to the mouse reference sequence (C57BL/6J, NCBI37/mm9) and subsequent variant calling resulted in a number of single nucleotide variants (SNVs) and insertions/deletions (INDELs) ranging from approximately 8,000 (C57BL/6J background) to over 200,000 (for more divergent strain backgrounds) variant calls per mutant exome, depending on strain background and depth of coverage. Generally, approximately two-thirds of the variants called were SNVs, rather than INDELS. However, in mutants on the C57BL/6J background, this ratio was closer to approximately one-half (Additional file 3). This is not surprising given that a large proportion of false positive calls from reference guided assembly are INDELs and the number of true variants in any C57BL/6J exome is expected to be low because the mouse reference strain is, primarily, C57BL/6J. The one exception was mutant 12860 (*nert*), which was reported to be on a C57BL/6J background; however, the relatively large number of variants detected in this mutant exome could indicate that the reported strain background is likely incorrect.

### Variant annotation and nomination of candidate mutations

The variant data were fully annotated according to genomic position, SNV quality, allele ratio (number of reads containing variant allele/number of reads containing reference allele), and overlap with current genome annotations, including NCBI Reference Sequence (RefSeq)/Ensembl genes, exons, introns, splice sites, and known SNVs, INDELs (the Single Nucleotide Polymorphism database, dbSNP). In each case, existing linkage data were used to determine map positions and the analysis was then limited to those regions. The existing linkage data ranged from coarse (chromosomal linkage) to fine (regions of < 10 to 20 Mb) (Additional file 3). The most likely causative mutations for each mutant sample and for a control C57BL/6J exome were nominated using the annotations as shown in Table 3. Specifically, novel (when compared to dbSNP) protein coding or splice site variants falling within mapped regions, with expected allele ratios (> 0.95 for homozygous variants and > 0.2 for heterozygous variants) were given priority for validation by re-sequencing of additional mutant and unaffected samples. To further reduce the validation burden, we found that comparison of unrelated exome sequencing data sets and comparison to the Sanger Institute Mouse Genomes data [16] allowed for significant reduction in validation burden, as any variants common between these data sets represent common variants that are shared between related strains or systematic false positives arising from mapping the data back to the reference sequence. Similar to what has been observed in human exome sequencing, the latter can be caused by repetitive or closely related sequences (paralogs) or underlying deficiencies in the reference sequence. For comparison, the alignment data from the C57BL/6J beta exome shown in Table 1 were subjected to variant calling and annotation. Interestingly, 17 variants passed filters in a C57BL/6J exome (Table 3), expected to be most similar to the reference genome, which is also primarily C57BL/6J. Comparison of these variants with the high throughput sequencing data for 17 inbred strains available from Sanger Mouse Genomes Project revealed three exonic SNVs unique to the C57BL/6J exome. We predict that the remaining 14 variants calls are false positive calls due to mapping errors, which can arise in regions where there is underlying deficiency in the reference sequence or in regions that share sequence similarity (that is, paralogs). These regions are apparent when viewing alignments as regions that contain a preponderance of non-uniquely mapped reads, gaps, or regions that contain apparent heterozygosity in samples that are known to be homozygous (as is the case with the inbred strain data from the Sanger Mouse Genomes project, where each strain was subjected to at least 200 generations of brother × sister intercrossing prior to sequencing; Additional file 5).

**Table 3:** Analysis of annotated variant data from mutant exome sequencing

Mutant number (allele)	Inheritance/phenotype	Mutation type: strain background	Variants called	In gene (introns, exons)	**Novel SNVs**^**a**^	Overlap with map position	**Allele ratio**^**b**^	Non-synonymous coding variants, splice sites	**Unique**^**c**^	Putative mutation
12874 (*bloodline*)	Recessive/metabolic	Spontaneous: stock (mixed B6)	134,205	116,120	35,469	350	155	29	1	*Map3k11*, E293K
12724 (*Cleft*)	Dominant/craniofacial	ENU: C57BL/6J, C3HeB/FeJ	49,367	36,037	10,873	83	53	19	2	*Col2a1*, Q713Stop
*repro7*	Recessive/reproductive	ENU: C57BL/6J, C3H/HeJ, Cast/EiJ	410,333	185,999	87,568	799	47	7	1	*Prdm9*, Q478Stop
5330 (*hpbk*)	Recessive/skeletal	ENU: C57BL/6J	8,516	6,167	4,589	35	3	2	2	*Notch3*, splice donor site (G to A), intron 31
13716 (*vgim*)	Recessive/reproductive	Spontaneous: C57BL/6J	10,134	7,346	5,533	117	6	3	2	*Lhfpl2*, G102E
8568 (*lear*)	Recessive/small ears	Spontaneous: C57BL/6J	8,219	5,715	1,889	12	1	1	1	*Prkra*, intron 5, splice donor
12856 (*shep*)	Recessive/metabolic	Spontaneous: A/J	164,116	59,067	16,930	454	177	83	1	*Relb*, Q334K
*l11Jus74*	Recessive	ENU: B6, 129	230,896	52,628	14,448	344	37	4	2	*Rundc3a*, Y46F; *Nek8*, V343E
4235 (*Sofa*)	Dominant, craniofacial	Spontaneous: C57BL/6J, AKR/J	134,207	116,122	35,471	346	310	121	1	*Pfas*, H1194_G1198del
*C57BL/6J*	NA	None	5,980	3,953	3,132	NA	538	17	3	NA
13716 (*vgim*)	Recessive/reproductive	Spontaneous: C57BL/6J	10,134	7,346	5,533	NA	940	97	38	NA

^a^Compared to dbSNP. ^b^> 0.95 for homozygous samples, > 0.2 for heterozygous samples. ^c ^compared to unrelated exome data sets. NA, not available.

### Validation of putative causative mutations

**Figure 2:**
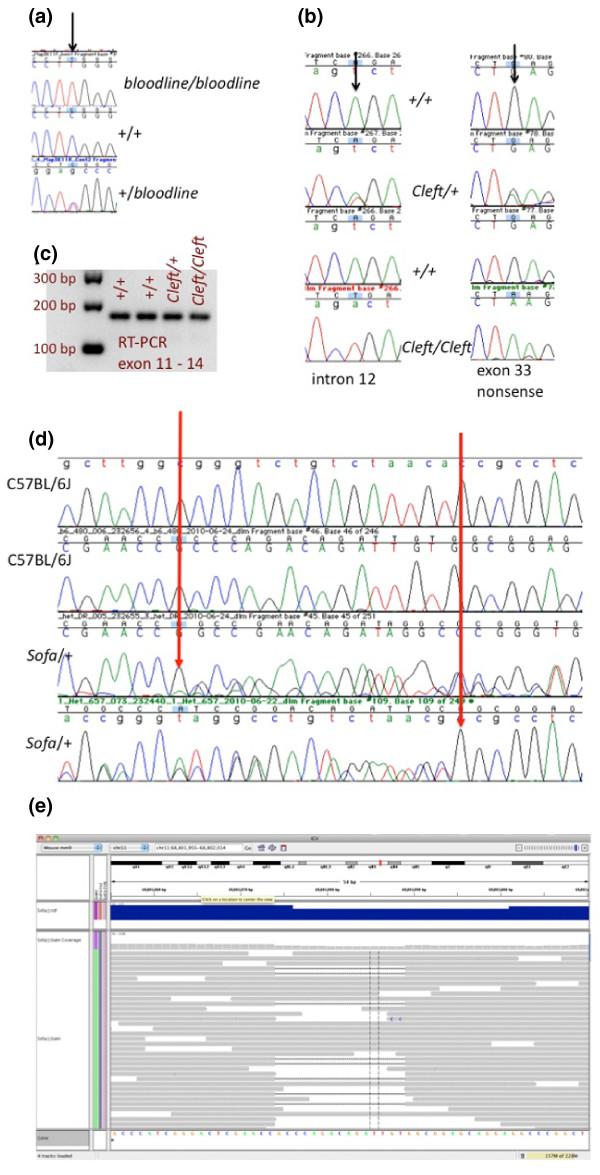
**Examples of validated mutations discovered in mutant exome data **. The *bloodline *mutation is a recessive mutation that causes a distinctive dorsal epidermal defect and tooth pulp necrosis. Exome sequencing revealed a G to A mutation in *Map3K11 *(*mitogen-activated protein kinase kinase kinase 11*). **(a) **PCR and sequencing of additional mutant (*bloodline/bloodline*) and unaffected (+/+ or +/-) animals provided additional support for this putative mutation. The '*Cleft*' mutation is an ENU mutation that arose on C57BL/6J. The mutation causes a dominant craniofacial phenotype and recessive perinatal lethality with characteristic cleft palate. **(b) **Sanger sequencing confirmed the presence of two closely linked mutations in multiple *cleft*/+ and *cleft/cleft *samples and the absence of these mutations in +/+ littermate samples. **(c) **Of the two mutations found, the intron mutation has the potential to cause splicing defects, although it is less likely to contribute to the phenotype since RT-PCR shows no indication of defective splicing mutant samples. The '*Sofa*' mutation is a spontaneous mutation that arose on C57BL/6J, causing a dominant craniofacial phenotype and recessive perinatal lethality. **(d) **Sanger sequencing of heterozygous and control samples confirmed the presence of a 15-bp deletion in *Pfas, FGAR amidotransferase*. **(e) **Reads from the mutant, deletion-bearing allele successfully mapped to *Pfas *using BWA (Burrows-Wheeler aligment tool) and the deletion was called using SAMtools [25] with an allele ratio of 0.2.

Using this approach, only one or two variants were nominated for validation in each of nine mutant exomes. Four of these mutants represented ENU-generated lines, while five were spontaneous mutants. In a few cases, the single variant nominated for validation proved to be the likely causative mutation. For example, the single SNV nominated for validation in the *bloodline *mutant correlated with the phenotype when additional affected and unaffected samples were tested (Figure 2a). The SNV is a missense mutation causing an amino acid change (E293K) in *Map3K11*, a gene that encodes a mitogen-activated protein kinase kinase kinase that is involved in a variety of cellular signaling cascades. Importantly, mice homozygous for a targeted null mutation in *Map3k11 *have the characteristic epidermal midline defect that is also observed in *bloodline *homozygotes [17], further implicating the missense mutation found as the causative mutation. Unlike *bloodline *homozygotes, *Map3K11*-/- mice are viable and tooth pulp necrosis has not been reported [17], indicating that the spontaneous mutation may be sensitive to strain background effects. However, further work is needed to establish the underlying mechanisms influencing these phenotypic differences.

In some cases, more than one potentially damaging variant was found to correlate with the phenotype when additional affected and unaffected animals from the pedigree were genotyped (Table 3). In two cases, *hpbk *and *vgim*, where more than one variant was found, only one variant could be validated while the other variants were false positives. In two cases where more than one potentially damaging variant was found, both were validated. Not surprisingly, these cases were ENU-induced mutant exomes (*Cleft *and *l11Jus74*) and ENU is known to cause mutations at a rate of greater than 1 in 750 per locus per gamete [18] at doses of 85 mg/kg. *Cleft *is a dominant craniofacial ENU mutation that causes cleft palate. Of the two variants that were nominated for validation, both were SNVs residing in *Col2a1*, a gene coding for type II procollagen. Both SNVs reside within 10 kb of each other (Chr15:97815207 and Chr15:97825743) in *Col2a1*, a gene coding for type II procollagen, and not surprisingly were found to be concordant with the phenotype when multiple animals from the pedigree were genotyped. The most likely causative lesion (G to A at Chr15:97815207) is a nonsense mutation that introduces a premature stop codon at amino acid 645. The second closely linked variant is an A to T transversion in intron 12 that could potentially act as a cryptic splice site. However, since RT-PCR did not reveal splicing abnormalities, it is more likely that the nonsense mutation is the causative lesion (Figure 2b). Mice homozygous for targeted deletions in *Col2a1 *and mice homozygous for a previously characterized, spontaneous mis-sense mutation, *Col2a1*^*sedc*^, share similar defects in cartilage development to *Cleft *mutants, including recessive peri-natal lethality and orofacial clefting [19,20], providing further support that the *Cleft *phenotype is the result of a mutation in *Col2a1*.

The *l11Jus74 *mutation was isolated in a screen for recessive lethal alleles on mouse chromosome 11 using a 129.*Inv*(*11*)*8Brd*^*Trp53-Wnt3 *^balancer chromosome [21,22]. The screen was performed as described previously using C57BL/6J ENU-treated males, mated to the balancer, which was generated in 129S5SvEv embryonic stem cells. Embryos from the *l11Jus74 *line were analyzed from timed matings, as previously described [23], to determine that homozygotes die perinatally. Two potentially causative missense mutations were found in *Nek8 *(NIMA (never in mitosis gene a)-related expressed kinase 8; V343E) and *Rundc3a *(Run domain containing 3a; Y46F). Mutations in *Nek8 *cause polycystic kidney disease, but no phenotypes have been ascribed to mutations in *Rundc3a*. Although the cause of death of *l11Jus74 *homozygotes has not been determined, polycystic kidneys have not been observed, making the most likely lesion to result in perinatal death *Rundc3a*, although the *Nek8 *mutation may cause a delayed onset phenotype.

For all four of the ENU-induced mutant exomes sequenced, putative causative mutations were nominated and validated. Mutations induced by ENU are usually single nucleotide substitutions. The high sensitivity of current analytical pipelines for detecting single nucleotide substitutions (and particularly homozygous substitutions), combined with the propensity of damaging single nucleotide substitutions to occur in coding sequences, likely explains the high success rate of exome sequencing for detecting induced lesions. Similarly, Boles *et al*. [24] showed that targeted sequencing of exons and highly conserved sequences from ENU mutants mapping to chromosome 11 yielded a high success rate, with candidate mutations nominated in nearly 75% of mutants.

While mutations induced by mutagens like ENU are known to cause single nucleotide substitutions, spontaneous mutations are the result of a variety of lesions, including single nucleotide substitutions, small INDELS and larger deletions or insertions of mobile DNA elements. Of the nine potentially damaging coding or splicing mutations discovered in this set of mutant exomes, the spontaneous *Sofa *mutant was the only one for which a single nucleotide substitution was not discovered. Instead, a 15-bp deletion in *Pfas *(Table 3; Figure 2d,e) was found, demonstrating that small deletions in coding sequence can be discovered using this approach.

Interestingly, the allele ratio for the *Sofa *deletion was 0.2, which is lower than expected for a heterozygote; therefore, a stringent cutoff of 0.5 or even 0.35, which we previously found was sufficient for calling heterozygous variants at approximately 80% confidence [4], would have eliminated this variant from consideration. The lower allele ratio is likely the result of bias in either the capture of the INDEL-containing fragments, and/or the ability to appropriately map some of the INDEL-bearing reads. Since the library fragments are larger than both the probes and the exons they target and because each target is tiled with multiple probes, there are expected to be perfect match probes somewhere within an exon for nearly every allele despite the presence of an INDEL. Consequently, we favor a mapping problem as the major driver for the lower than expected allele ratio observed (Figure 2e). Longer reads may alleviate some systematic issues associated with discovering relevant deletions or insertions. A 15-bp deletion would maximally comprise a mismatch of nearly 38% along a 40-bp read, but only 20% within a 76-bp read. Large gaps (20% or more of the read) would impose a stiff mapping penalty on that end of read pairs. Presumably, longer reads (100 bp or longer) would incur lower penalties, thereby moderating adverse mapping effects.

Approximately 10% of known deleterious mutations in the mouse genome affect the conserved splice acceptor or donor sites (Table 4), which include the two intronic nucleotides immediately flanking each exon. Of the putative mutations discovered in this set of 15 mutant exomes, three candidates were found in or immediately adjacent to the conserved splice acceptor or donor sites (*Cleft, lear*, and *hpbk*), demonstrating that exome sequencing provides sufficient coverage of flanking intron sequence to positively identify potentially damaging, non-coding mutations in the intron sequences immediately flanking target exons.

**Table 4:** *In silico *analysis of all induced or spontaneous alleles (4,984) with phenotypes reported in the Mouse Genomes Database [1]

Mutation	Number of alleles
Unknown or uncharacterized	3,105
Introns, UTRs, regulatory regions (including instances where the lesion is not known but coding sequence has been sequenced), cryptic splice sites, inversions	150
Exons (single nucleotide substitutions, deletions, insertions)	1,581
Conserved splice acceptor or donor	148

This analysis shows that the vast majority of induced or spontaneous alleles that have been characterized at the molecular level (1,879) are mutations in coding sequence or conserved splice acceptor/splice donor sites.

### Traditional genetic mapping and exome sequencing

In all cases, either coarse mapping data (chromosomal linkage) or a fine map position (< 20 Mb) was available to guide analysis and ease validation burden (Additional file 3). For example, the *shep *mutation was previously linked to chromosome 7 (approximately 152 Mb), while *repro7 *was fine mapped to a 4.5 Mb region on chromosome 17. The mapping of *shep *to chromosome 7 was accomplished using a group of 20 affected animals, while the fine mapping of *repro7 *to a 4.5 Mb region on chromosome 17 required the generation of 524 F2 animals, requiring over a year of breeding in limited vivarium space. In both cases, the mapping data coupled with the additional filtering of annotated data, as shown in Table 3, significantly reduced the validation burden to a single variant. Therefore, high-throughput sequencing (exome or whole genome) represents a cost efficient alternative to fine mapping by recombination, especially in cases where vivarium space and time are limited resources.

In the absence of chromosomal linkage, the validation burden is significantly larger. For example, the *vgim *mutant exome was reanalyzed without utilizing mapping information (Table 3, last row) and 38 variants were nominated for validation. Addition of just the chromosomal linkage data for *vgim *(chromosome 13), but not the fine mapping data (chr13:85473357-96594659) reduces the validation burden to two candidates. Therefore, coarse mapping to establish chromosomal linkage provides significant reduction in validation burden at minimal additional animal husbandry cost and time. In the absence of mapping data and/or when mutations arise on unusual genetic backgrounds, exome sequencing of additional samples (affected animal and parents) would similarly reduce the validation burden to just one or a few variants.

### Limitations of exome sequencing for mutation discovery

Using this technology, we validated putative causative coding mutations in 9 of the 15 mutant exomes examined. For the remaining six mutants, candidate mutations were found in UTRs or were not found at all (Table 5). For *Alf, nert *and *aphl*, candidate mutations were found in UTRs, and interestingly, in nearly every case, these candidate mutations are in genes not currently associated with any mouse phenotype. For the other three mutants, *frg, stn *and *sunk*, no candidate mutations were found in protein coding sequence, splice sites or in UTRs. Failure to identify the candidate causative mutations most likely indicates that these mutations reside in non-coding, regulatory regions or unannotated coding sequence that is not included in the current exome capture design. An additional possibility is that the underlying mutations do reside in the targeted regions, but are simply not revealed using standard mapping and SNP calling, which is clearly biased towards the discovery of single nucleotide substitutions and small INDELs. Robust computational methods for finding larger insertions and deletions and/or translocations via high-throughput sequencing data are not widely available and the absence of these tools limits spontaneous mutation discovery by any means, whether exome or whole genome sequencing.

**Table 5:** Validation of putative causative coding mutations in 15 mutant exomes

Mutant number (allele)	Inheritance/phenotype	Strain background	Variants called	In gene (introns, exons)	**Novel SNVs**^**a**^	Overlap with map position	**Allele ratio**^**b**^	Non-synonymous coding variants, splice sites	**Unique**^**c**^	Validation of coding/splice variants	Variants in UTRs
*5413 *(*Plps*)	Dominant/craniofacial	Spontaneous: C57BL/6J, 129S1/SvImJ	13,453	3,271	1,821	200	129	55	3	None	3: *Kcnab3, Pigs, Accn1*
*12860 *(*nert*)	Recessive/craniofacial	Spontaneous: C57BL/6J	121,109	105,964	30,275	1,441	639	94	3	None	4: *4931406P16Rik, Shisa7, Nipa1, Alpk3*
*13782 *(*aphl*)	Recessive/skin, hair	Spontaneous: MRL/MpJ	182,564	156,802	57,317	554	366	33	1	None	4: *Eif2ak3, Mrpl35, Usp39 *(2)
*6246 *(*sunk*)	Recessive/size	Spontaneous: A/J	164,053	60,051	16,508	693	303	25	0	None	None
*3485 *(*frg*)	Recessive/craniofacial	Spontaneous: C57BL/6J, A/J	124,054	105,326	20,073	36	22	0	0	None	None
*4507 *(*stn*)	Recessive/craniofacial	Spontaneous: C57BL/6J	7,523	3,079	2,338	13	7	0	0	None	None

In 6 of the 15 mutant exomes sequenced, candidate mutations in protein coding sequence or splice sites were either not found or could not be validated in additional samples; for three of these, however, candidate mutations in regions annotated at UTRs were identified. ^a^Compared to dbSNP. ^b^> 0.95 for homozygous samples, > 0.2 for heterozygous samples. ^c^compared to unrelated exome data sets.

In a parallel effort, we used targeted sequencing of contiguous regions to discover spontaneous mutations that have been mapped to regions of 10 Mb or less. Interestingly, the success rate for nominating putative mutations via targeted sequencing of contiguous regions was comparable to that of exome sequencing (at approximately 60%), demonstrating that despite the availability of sequence data representing the entire candidate region, existing analysis pipelines are not sufficient for discovery of all disease-causative genetic lesions. Moreover, systematic errors in the mm9 reference sequence or insufficient gene annotation [24] are also likely to contribute to failed mutation discovery, since current analytical approaches rely upon reference and contemporary gene annotation as assumed underlying truth.

In this context, it is notable that the exome-based analysis of human phenotypes that are presumed to be monogenic is also frequently unsuccessful, although such negative results are generally not reported in the literature. Consequently, we anticipate that deeper analysis of the mouse mutants that fail discovery by exome sequencing may also shed light on the nature of both non-coding and cryptic coding mutations that contribute to Mendelian phenotypes in humans.

## Conclusions

Whole exome sequencing is a robust method for mutation discovery in the mouse genome and will be particularly useful for high-throughput genetic analyses of large mutant collections. Due to the nature of the underlying mutations and the current methods available for massively parallel sequence data analysis, ENU mutation discovery via exome sequencing is more successful than spontaneous mutation discovery. In all cases, coarse mapping data (chromosomal linkage) significantly eased validation burden (Table 3); however, fine mapping to chromosomal regions < 10 to 20 Mb, while useful, did not provide significant added value (Table 3; Additional file 3). A similar conclusion was drawn by Arnold *et al*. [5] for mutation discovery via whole genome sequencing. In addition, since the data shown here include mutations on a variety of strain backgrounds, comparison across unrelated exome data sets and to whole genome sequencing data from the Mouse Genomes Project [16] proved critical in reducing the validation burden, especially where mapping data were not available to guide analysis.

Although we are 10 years past the assembly of both the human and mouse genomes, the biological function of the vast majority of mammalian genes remains unknown. We anticipate that the application of exome sequencing to the thousands of immediately available mutant mouse lines exhibiting clinically relevant phenotypes will make a large and highly valuable contribution to filling this knowledge gap.

## Materials and Methods

### Exome capture and sequencing

The following protocol for exome capture and sequencing is the standard protocol generally followed by all sites providing data for proof-of-concept experiments. Site-specific deviations in the standard protocol can be provided upon request. The mouse exome probe pools developed in this study, SeqCap EZ Mouse Exome SR, are commercially available on request from Roche NimbleGen.

#### DNA extraction

DNA for high-throughput sequencing was isolated from spleen using a Qiagen DNeasy Blood and Tissue kit (Qiagen, Santa Clarita, CA USA) or by phenol/chloroform extraction of nuclear pellets. Briefly, spleen samples were homogenized in ice-cold Tris lysis buffer (0.02 M Tris, pH 7.5, 0.01 M NaCl, 3 mM MgCl_2_). Homogenates were then incubated in 1% sucrose, 1% NP40 to release nuclei, which were subsequently pelleted by centrifugation at 1,000 rpm, 4°C. Isolated nuclei were then extracted by phenol chloroform in the presence of 1% SDS. DNA for PCR was extracted from small (1 to 2 mm) tail biopsies by lysing in 200 ml of 50 mM NaOH at 95°C for 10 minutes. Samples were neutralized by adding 20 ml of 1 M Tris HCl, pH 8.0 and used directly for PCR amplification.

#### Capture library preparation and hybridization amplification

Illumina PE libraries (Illumina, San Diego, CA, USA) were constructed using Illumina's Multiplexing Kit (part number PE-400-1001) with a few modifications. Size selection was done using the Pippin Prep from Sage Science, Inc. (Beverly, MA, USA). The target base pair selection size was set at 430 bp. The entire 40 μl recovery product was used as template in the pre-hybridization library amplification (using ligation-mediated PCR (LMPCR)). Pre-hybridization LMPCR consisted of one reaction containing 50 μl Phusion High Fidelity PCR Master Mix (New England BioLabs, Ipswich, MA, USA; part number F-531L), 0.5 μM of Illumina Multiplexing PCR Primer 1.0 (5'-AATGATACGGCGACCACCGAGATCTACACTCTTTCCCTACACGACGCTCTTCCGATCT-3'), 0.001 μM of Illumina Multiplexing PCR Primer 2.0 (5'-GTGACTGGAGTTCAGACGTGTGCTCTTCCGATCT-3'), 0.5 μM of Illumina PCR Primer, Index 1 (or other index at bases 25-31; 5'-CAAGCAGAAGACGGCATACGAGAT(CGTGATG)TGACTGGAGTTC-3'), 40 μl DNA, and water up to 100 μl. PCR cycling conditions were as follows: 98°C for 30 s, followed by 8 cycles of 98°C for 10 s, 65°C for 30 s, and 72°C for 30 s. The last step was an extension at 72°C for 5 minutes. The reaction was then kept at 4°C until further processing. The amplified material was cleaned with a Qiagen Qiaquick PCR Purification Kit (part number 28104) according to the manufacturers instructions, except the DNA were eluted in 50 μl of water. DNA was quantified using the NanoDrop-1000 (Wilmington, DE, USA) and the library was evaluated electrophoretically with an Agilent Bioanalyzer 2100 (Santa Clara, CA, USA) using a DNA1000 chip (part number 5067-1504). Sample multiplexing was performed in some cases, after capture and prior to sequencing.

#### Liquid phase sequence capture and processing

Prior to hybridization the following components were added to a 1.5 ml tube: 1.0 μg of library material, 1 μl of 1,000 μM oligo 5'- AATGATACGGCGACCACCGAGATCTACACTCTT TCCCTACACGACGCTCTT CCG ATC*T-3' (asterisk denotes phosphorothioate bond), 1 μl of 100 μM oligo 5' CAAGCAGAAGACGGCATACGAGATCGTGATGTGACTGGAGTTCAGACGTGTGCTCTTCCGATC*T-3' (bases 25 to 31 correspond to index primer 1), and 5 μg of Mouse COT-1 DNA (part number 18440-016; Invitrogen, Inc., Carlsbad, CA, USA). Samples were dried down by puncturing a hole in the 1.5-ml tube cap with a 20 gauge needle and processing in an Eppendorf Vacufuge (San Diego, CA, USA) set to 60°C for 20 minutes. To each sample 7.5 μl NimbleGen SC Hybridization Buffer (part number 05340721001) and 3.0 μl NimbleGen Hybridization component A (part number 05340721001) were added, sample was vortexed for 30 s, centrifuged, and placed in a heating block at 95°C for 10 minutes. The samples were again mixed for 10 s, and spun down. This mixture was then transferred to a 0.2-ml PCR tube containing 4.5 μl of Mouse Exome Solution Phase probes and mixed by pipetting up and down ten times. The 0.2 ml PCR tubes were placed in a thermocylcer with heated lid at 47°C for 64 to 72 hours. Washing and recovery of captured DNA were performed as described in chapter 6 of the NimbleGen SeqCap EZ Exome SR Protocol version 2.2 (available from the Roche NimbleGen website) [11]. Samples were then quality checked using quantitative PCR as described in chapter 8 of the SR Protocol version 2.2 [10]. Sample enrichment was calculated and used as a means of judging capture success. Mean fold enrichment greater than 50 was considered successful and sequenced. NimbleGen Sequence Capture Control (NSC) quantitative PCR assay NSC-0272 was not used to evaluate captures in these experiments.

#### Post-hybridization LMPCR

Post-hybridization amplification (for example, LMPCR via Illumina adapters) consisted of two reactions for each sample using the same enzyme concentration as the pre-capture amplification, but a modified concentration, 2 uM, and different versions of the Illumina Multiplexing 1.0 and 2.0 primers were employed: forward primer 5'- AATGATACGGCGACCACCGAGA and reverse primer 5'-CAAGCAGAAGACGGCATACGAG. Post-hybridization amplification consisted of 16 cycles of PCR with identical cycling conditions as used in the pre-hybridization LMPCR (above), with the exception of the annealing temperature, which was lowered to 60°C. After completion of the amplification reaction, the samples were purified using a Qiagen Qiaquick column following the manufacturer's recommended protocol. DNA was quantified spectrophotometrically, and electrophoretically evaluated with an Agilent Bioanalyzer 2100 using a DNA1000 chip (Agilent). The resulting post-capture enriched sequencing libraries were diluted to 10 nM and used in cluster formation on an Illumina cBot and PE sequencing was done using Illumina's Genome Analyzer IIx or Illumina HiSeq. Both cluster formation and PE sequencing were performed using the Illumina-provided protocols.

### High-throughput sequencing data analysis

#### Mapping, SNP calling and annotation

The sequencing data were mapped using Maq, BWA (Burrows-Wheeler alignment tool) and/or GASSST (global alignment short sequence search tool) and SNP calling was performed using SAMtools [25] and/or GenomeQuest [26]. SNP annotation was performed using GenomeQuest, custom scripts and Galaxy tools. Alignments were visualized with the UCSC genome browser, Integrated Genomics Viewer (Broad Institute) and/or SignalMap (Roche NimbleGen).

#### Validation

Candidate mutations were validated by PCR amplification and sequencing of affected and unaffected samples if available from the mutant colony or from archived samples. Sequencing data were analyzed using Sequencher 4.9 (Gene Codes Corp., Ann Arbor, MI, USA). Primers were designed using Primer3 software [27].

#### RT-PCR

Total RNA was isolated from heterozygous and homozygous tail biopsies and/or embryos using the RNeasy Mini Kit (Qiagen) according to the manufacturer's protocols. Total RNA (1 μg) was reverse transcribed into cDNA using the SuperScript III First-Strand Synthesis SuperMix for quantitative RT-PCR (Invitrogen) according to the manufacturer's protocols. cDNA (3 μl) was used as template in a 30 μl PCR with the following cycling conditions for all primers (0.4 μM final concentration): 94°C (45 s), 56°C (45 s), 72°C (45 s) for 30 cycles. Primers used for *Cleft *were Cleft_11-14f (5'-CTGGAAAACCTGGTGACGAC) and Cleft_11-14R (5'-ACCAGCTTCCCCCTTAGC).

## Abbreviations

bp: base pair; dbSNP: Single Nucleotide Polymorphism Database; ENU: *N*-ethyl-*N*-nitrosourea; INDEL: insertions/deletion; LMPCR: ligation-mediated PCR; NCBI: National Center for Biotechnology Information; PCR: polymerase chain reaction; PE: paired-end; RefSeq: NCBI Reference Sequence; RT-PCR: reverse transcriptase polymerase chain reaction; SNV: single nucleotide variant; UTR: untranslated region; VEGA: The Vertebrate Genome Annotation database.

## Competing interests

The authors from Roche NimbleGen recognize a competing interest in this publication as employees of the company. The other authors declare that they have no competing interests.

## Authors' contributions

JJ, LGR, JS, BTK, IG, JH, and SWL participated in the conception of the mouse exome design. CB and JR created and provided the gene list that was the basis for the exome design. JS, SS, EM, FDP, and KLT provided sequencing support. MSS, LGR, SAM, LRD, DEB, MLC, TCC, and SWL provided mutant samples. WH, CH, DG, HF, GG, MB, LR, RRC, FJP, and MC performed sample preparation, exome capture, PCR and RT-PCR validation. YD, MD, DG, and TR provided sequence analysis and bioinformatics support. LGR, JS and JJ conceived of the study, and participated in its design and coordination and drafted the manuscript. All authors read and approved the final manuscript.

## Supplementary Material

Additional file 1 **Summary statistics for the alpha and beta exome probe pools**. Click here for file

Additional file 2 **Comparison of 2 × 76-bp datasets from four independent captures of female C56BL/6J DNA and one capture of male C57BL/6J compared to alpha data from one capture of male C57BL/6J**. Click here for file

Additional file 3 **Additional data on mutant exomes sequenced in this study**. Genetic background, size of mapped intervals, genotype of sequenced sample and percentage of SNVs identified are provided. Click here for file

Additional file 4 **Data generated from exome sequencing of mutant and control exomes (2 × 40 bp, 2 × 76 Illumina or 2 × 100 HiSeq)**. Click here for file

Additional file 5 **Seventeen variants passing filter in a C57BL/6J exome**. The genome coordinate and gene annotation for each variant are provided. Comparison of these variants with the high-throughput sequencing data for 17 inbred strains available from Sanger Mouse Genomes Project revealed three exonic SNVs that are likely unique to the C57BL/6J exome. Click here for file

## References

[B1] Mouse Genome Informatics. http://www.informatics.jax.org/mgihome/homepages/stats/all_stats.shtml

[B2] Shendure J Ji H Next-generation DNA sequencing. Nat Biotechnol 2008 26 1135 1145 10.1038/nbt1486 18846087

[B3] Zhang Z Alpert D Francis R Chatterjee B Yu Q Tansey T Sabol SL Cui C Bai Y Koriabine M Yoshinaga Y Cheng JF Chen F Martin J Schackwitz W Gunn TM Kramer KL De Jong PJ Pennacchio LA Lo CW Massively parallel sequencing identifies the gene Megf8 with ENU-induced mutation causing heterotaxy. Proc Natl Acad Sci USA 2009 106 3219 3224 10.1073/pnas.0813400106 19218456 PMC2651267

[B4] D'Ascenzo M Meacham C Kitzman J Middle C Knight J Winer R Kukricar M Richmond T Albert TJ Czechanski A Donahue LR Affourtit J Jeddeloh JA Reinholdt L Mutation discovery in the mouse using genetically guided array capture and resequencing. Mamm Genome 2009 20 424 436 10.1007/s00335-009-9200-y 19629596 PMC2829192

[B5] Arnold CN Xia Y Lin P Ross C Schwander M Smart NG Muller U Beutler B Rapid identification of a disease allele in mouse through whole genome sequencing and bulk segregation analysis. Genetics 2011 187 633 641 10.1534/genetics.110.124586 21196518 PMC3063661

[B6] Ng SB Bigham AW Buckingham KJ Hannibal MC McMillin MJ Gildersleeve HI Beck AE Tabor HK Cooper GM Mefford HC Lee C Turner EH Smith JD Rieder MJ Yoshiura K Matsumoto N Ohta T Niikawa N Nickerson DA Bamshad MJ Shendure J Exome sequencing identifies MLL2 mutations as a cause of Kabuki syndrome. Nat Genet 2010 42 790 793 10.1038/ng.646 20711175 PMC2930028

[B7] Ng SB Buckingham KJ Lee C Bigham AW Tabor HK Dent KM Huff CD Shannon PT Jabs EW Nickerson DA Shendure J Bamshad MJ Exome sequencing identifies the cause of a mendelian disorder. Nat Genet 2010 42 30 35 10.1038/ng.499 19915526 PMC2847889

[B8] Zuchner S Dallman J Wen R Beecham G Naj A Farooq A Kohli MA Whitehead PL Hulme W Konidari I Edwards YJ Cai G Peter I Seo D Buxbaum JD Haines JL Blanton S Young J Alfonso E Vance JM Lam BL Pericak-Vance MA Whole-exome sequencing links a variant in DHDDS to retinitis pigmentosa. Am J Hum Genet 2011 88 201 206 10.1016/j.ajhg.2011.01.001 21295283 PMC3035708

[B9] Ostergaard P Simpson MA Brice G Mansour S Connell FC Onoufriadis A Child AH Hwang J Kalidas K Mortimer PS Trembath R Jeffery S Rapid identification of mutations in GJC2 in primary lymphoedema using whole exome sequencing combined with linkage analysis with delineation of the phenotype. J Med Genet 2011 48 251 255 10.1136/jmg.2010.085563 21266381

[B10] Walsh T Shahin H Elkan-Miller T Lee MK Thornton AM Roeb W Abu Rayyan A Loulus S Avraham KB King MC Kanaan M Whole exome sequencing and homozygosity mapping identify mutation in the cell polarity protein GPSM2 as the cause of nonsyndromic hearing loss DFNB82. Am J Hum Genet 2010 87 90 94 10.1016/j.ajhg.2010.05.010 20602914 PMC2896776

[B11] Bainbridge MN Wang M Burgess DL Kovar C Rodesch MJ D'Ascenzo M Kitzman J Wu YQ Newsham I Richmond TA Jeddeloh JA Muzny D Albert TJ Gibbs RA Whole exome capture in solution with 3 Gbp of data. Genome Biol 2010 11 R62 10.1186/gb-2010-11-6-r62 20565776 PMC2911110

[B12] Choi M Scholl UI Ji W Liu T Tikhonova IR Zumbo P Nayir A Bakkaloglu A Ozen S Sanjad S Nelson-Williams C Farhi A Mane S Lifton RP Genetic diagnosis by whole exome capture and massively parallel DNA sequencing. Proc Natl Acad Sci USA 2009 106 19096 19101 10.1073/pnas.0910672106 19861545 PMC2768590

[B13] Blake JA Bult CJ Kadin JA Richardson JE Eppig JT The Mouse Genome Database (MGD): premier model organism resource for mammalian genomics and genetics. Nucleic Acids Res 2011 39 D842 848 10.1093/nar/gkq1008 21051359 PMC3013640

[B14] Mouse Exome Gene List. ftp://ftp.jax.org/Genome_Biology_mouse_exomes/mouse_exome_genes.xls.zip

[B15] Mouse Exome Design. ftp://ftp.jax.org/Genome_Biology_mouse_exomes/100803_MM9_exome_rebal_2_EZ_HX1.gff.bz2

[B16] Mouse Genomes Project. http://www.sanger.ac.uk/resources/mouse/genomes/

[B17] Brancho D Ventura JJ Jaeschke A Doran B Flavell RA Davis RJ Role of MLK3 in the regulation of mitogen-activated protein kinase signaling cascades. Mol Cell Biol 2005 25 3670 3681 10.1128/MCB.25.9.3670-3681.2005 15831472 PMC1084312

[B18] Hitotsumachi S Carpenter DA Russell WL Dose-repetition increases the mutagenic effectiveness of N-ethyl-N-nitrosourea in mouse spermatogonia. Proc Natl Acad Sci USA 1985 82 6619 6621 10.1073/pnas.82.19.6619 3863118 PMC391261

[B19] Leung AW Wong SY Chan D Tam PP Cheah KS Loss of procollagen IIA from the anterior mesendoderm disrupts the development of mouse embryonic forebrain. Dev Dyn 2010 239 2319 2329 10.1002/dvdy.22366 20730911

[B20] Donahue LR Chang B Mohan S Miyakoshi N Wergedal JE Baylink DJ Hawes NL Rosen CJ Ward-Bailey P Zheng QY Bronson RT Johnson KR Davisson MT A missense mutation in the mouse Col2a1 gene causes spondyloepiphyseal dysplasia congenita, hearing loss, and retinoschisis. J Bone Miner Res 2003 18 1612 1621 10.1359/jbmr.2003.18.9.1612 12968670 PMC2862909

[B21] Kile BT Hentges KE Clark AT Nakamura H Salinger AP Liu B Box N Stockton DW Johnson RL Behringer RR Bradley A Justice MJ Functional genetic analysis of mouse chromosome 11. Nature 2003 425 81 86 10.1038/nature01865 12955145

[B22] Zheng B Sage M Cai WW Thompson DM Tavsanli BC Cheah YC Bradley A Engineering a mouse balancer chromosome. Nat Genet 1999 22 375 378 10.1038/11949 10431243

[B23] Hentges KE Nakamura H Furuta Y Yu Y Thompson DM O'Brien W Bradley A Justice MJ Novel lethal mouse mutants produced in balancer chromosome screens. Gene Expr Patterns 2006 6 653 665 10.1016/j.modgep.2005.11.015 16466971

[B24] Boles MK Wilkinson BM Wilming LG Liu B Probst FJ Harrow J Grafham D Hentges KE Woodward LP Maxwell A Mitchell K Risley MD Johnson R Hirschi K Lupski JR Funato Y Miki H Marin-Garcia P Matthews L Coffey AJ Parker A Hubbard TJ Rogers J Bradley A Adams DJ Justice MJ Discovery of candidate disease genes in ENU-induced mouse mutants by large-scale sequencing, including a splice-site mutation in nucleoredoxin. PLoS Genet 2009 5 e1000759 10.1371/journal.pgen.1000759 20011118 PMC2782131

[B25] Galaxy. http://main.g2.bx.psu.edu

[B26] GenomeQuest. http://www.genomequest.com/

[B27] Primer3. http://frodo.wi.mit.edu/primer3/

[B28] Mouse Mutant Re-sequencing Project. http://www.broadinstitute.org/scientific-community/science/projects/mammals-models/mouse/mouse-mutant-resequencing

