# Supplementary material for: Mutation discovery in mice by whole exome sequencing
Source: Genome Biol. 2011 Sep 14;12(9):R86. doi: 10.1186/gb-2011-12-9-r86 (PMC3308049; doi:10.1186/gb-2011-12-9-r86)
Supplement: Additional file 2 — Comparison of 2 × 76-bp datasets from four independent captures of female C56BL/6J DNA and one capture of male C57BL/6J compared to alpha data from one capture of male C57BL/6J. [file gb-2011-12-9-r86-S2.DOCX]

**Additional File 2.** **Comparison of 2 x 76 bp datasets from four independent captures of female C56BL/6J DNA and one capture of male C57BL/6J compared to alpha data from one capture of male C57BL/6J.**

| Sample | **C57BL/6J male** | | **C57BL/6J female** | **C57BL/6J female** | **C57BL/6J female** | **C57BL/6J female** |
| --- | --- | --- | --- | --- | --- | --- |
| Design | Alpha | Beta | Beta | | | |
| Percent Bases Covered | 97.4 | 98.5 | 97.9 | 97.8 | 97.9 | 97.9 |
| Percent Bases Not Covered | 2.6 | 1.5 | 2.1 | 2.2 | 2.1 | 2.1 |
| Coverage at 20X | 69.6 | 81.2 | 79.7 | 80.9 | 80.7 | 78.2 |
| Coverage at 10X | 84.9 | 92.5 | 90.9 | 91.3 | 91.3 | 90.5 |
| Coverage at 5X | 92.0 | 96.0 | 95 | 95.1 | 95.1 | 94.9 |
| Coverage at 1X | 97.4 | 98.5 | 97.9 | 97.8 | 97.9 | 97.9 |
| Total Reads | 54324684 | 127323572 | 65194138 | 68261190 | 67151848 | 61145512 |
| **2 x 76 bp, Illumina GAIIX |  |  |  |  |  |  |
