# Supplementary material for: Mutation discovery in mice by whole exome sequencing
Source: Genome Biol. 2011 Sep 14;12(9):R86. doi: 10.1186/gb-2011-12-9-r86 (PMC3308049; doi:10.1186/gb-2011-12-9-r86)
Supplement: Additional file 3 — Additional data on mutant exomes sequenced in this study. Genetic background, size of mapped intervals, genotype of sequenced sample and percentage of SNVs identified are provided. [file gb-2011-12-9-r86-S3.DOCX]

|  | **Genetic background** | **mapped region** | **Size of mapped region** | **Putative mutation found? (Y/N)** | **Genotype of sequenced sample** | **% SNVs (SNVs/total variants called)** |
| --- | --- | --- | --- | --- | --- | --- |
| *5413 (Plps)* | C57BL/6J, 129S1/SvImJ | chr11:60,325,202-76,777,433 | ~16 Mb | N | heterozygous | 71% |
| *12860 (nert)* | C57BL/6J | chr7:0-56764849 | ~56 Mb | N | homozygous | 82% |
| *13782 (aphl)* | MRL/MpJ | chr6 | ~150 Mb | N | homozygous | 78% |
| *6246 (sunk)* | A/J | chr14 | ~125 Mb | N | homozygous | 79% |
| *3485* *(frg)* | C57BL/6J, A/J | chr13:38,855,131-53,042,973 | ~14.2 Mb | N | homozygous | 89% |
| *4507* *(stn)* | C57BL/6J | chr19:20,420,342-54,969,161 | ~34.5 Mb | N | homozygous | 64% |
| *12874 (bloodline)* | Stock (mixed B6) | chr19:5,321,112-10,780,879 | ~5.4 Mb | Y | homozygous | 79% |
| *12724 (Cleft)* | C57BL/6J, C3HeB/FeJ | chr15:76,840,723-103396240 | ~26.2 Mb | Y | heterozygous | 64% |
| *repro7* | C57BL/6J, C3H/HeJ, Cast/EiJ | chr17:14650744-19281034 | ~4.5 Mb | Y | homozygous | 62% |
| *5330 (hpbk)* | C57BL/6J | chr17:25502885-37470737 | ~11.9 Mb | Y | homozygous | 44% |
| *13716 (vgim)* | C57BL/6J | chr13:85473357-96594659 | ~11.1 Mb | Y | homozygous | 44% |
| *8568 (lear)* | C57BL/6J | chr2:74,972,473-79,940,834 | ~5 Mb | Y | homozygous | 45% |
| *12856 (shep)* | A/J | chr 7 | ~152 Mb | Y | homozygous | 78% |
| *l11Jus74* | B6, 129 | chr11 | ~121 Mb | Y | homozygous | 70% |
| *4235 (Sofa)* | C57BL/6J, AKR/J | chr11:68,422,759-72,046,406 | ~3.6 Mb | Y | heterozygous | 79% |

**Additional File 4. Additional data on mutant exomes sequenced in this study. Genetic background, size of mapped intervals, genotype of sequenced sample and % SNVs identified are provided.**
